# The Efficacy and Safety of the Addition of Mitoxantrone Hydrochloride Liposome in Conditioning Regimen for High‐Risk Acute Myeloid Leukemia

**DOI:** 10.1002/hon.70116

**Published:** 2025-07-03

**Authors:** Xiaoyu Zhang, Donglin Yang, Aiming Pang, Sizhou Feng, Mingzhe Han, Yi He, Erlie Jiang

**Affiliations:** ^1^ State Key Laboratory of Experimental Hematology Haihe Laboratory of Cell Ecosystem National Clinical Research Center for Blood Diseases Institute of Hematology & Blood Diseases Hospital Chinese Academy of Medical Sciences & Peking Union Medical College Tianjin China; ^2^ Tianjin Institutes of Health Science Tianjin China

**Keywords:** acute myeloid leukemia (AML), allogeneic hematopoietic stem cell transplantation (allo‐HSCT), ELN high‐risk, mitoxantrone hydrochloride liposome

## Abstract

Despite allo‐HSCT being the primary curative treatment for high‐risk AML, relapse‐free survival (RFS) remains suboptimal due to high relapse incidence. Our research focuses on optimizing the conditioning regimen by incorporating Mitoxantrone Hydrochloride Liposome (Lipo‐MIT), a novel nano‐formulation with enhanced pharmacokinetic properties and demonstrated anti‐leukemic efficacy. Preclinical studies have shown that Lipo‐MIT significantly improves survival outcomes compared to conventional mitoxantrone, and our study aims to translate these findings into clinical practice. In this study, we present the results of a Lipo‐MIT as part of the conditioning regimen for high‐risk AML patients undergoing allo‐HSCT. Our findings highlight the potential of Lipo‐MIT to improve RFS, while also providing insights into patient selection and the refinement of Lipo‐MIT‐based conditioning strategies. We believe this work contributes valuable knowledge to the field and has the potential to impact clinical practice.

## Peer Review

The peer review history for this article is available at https://www.webofscience.com/api/gateway/wos/peer-review/10.1002/hon.70116.

To Editor,

The prognosis for high‐risk acute myeloid leukemia (AML) remains poor, with allogeneic hematopoietic stem cell transplantation (allo‐HSCT) being the primary curative treatment. However, relapse‐free survival (RFS) post‐transplantation remains low, mainly due to high relapse incidence [1]. To improve outcomes, optimizing the conditioning regimen to reduce relapse incidence is critical [2]. Mitoxantrone, a synthetic anthracycline agent, has shown significant anti‐leukemic activity in the treatment of hematology malignances, especially AML [3, 4]. Mitoxantrone hydrochloride liposome (Lipo‐MIT), a novel nano‐formulation, exhibits promising pharmacokinetic properties and improved survival outcomes in preclinical models compared to conventional mitoxantrone [5]. Previous clinical studies have consistently supported its anti‐tumor efficacy and safety profile [6, 7]. In this study, we evaluate the use of Lipo‐MIT as part of the conditioning regimen in high‐risk AMLs receiving allo‐HSCT, aiming to provide new insights for conditioning regimen refinements.

A total of 26 high‐risk AML patients, classified according to the European Leukemia Net (ELN) criteria, who underwent allo‐HSCT at the Institute of Hematology and Blood Diseases Hospital, Chinese Academy of Medical Sciences & Peking Union Medical College (CAMS & PUMC) between October 2022 and April 2024, were included in this study. This study was part of a larger study, the “NICHE‐BMT” project (NCT04645199), which was approved by the IHCAMS Clinical Research Academic Committee and the IHCAMS Ethics Committee on February 7, 2021 (IIT2021011‐EC‐1). All patients received intravenous liposomal mitoxantrone (Lipo‐MIT) at 30 mg/m^2^ on day −7, followed by fludarabine (30 mg/m^2^/day, days −6 to −2), busulfan (3.2 mg/kg/day, days −5 to −2), and cytarabine (2 g/m^2^/day, days −5 to −2). Neutrophil and platelet engraftment were defined using established criteria. Acute and chronic graft‐versus‐host disease (GVHD) were graded according to international standards. Relapse, non‐relapse mortality (NRM), RFS, and overall survival (OS) were assessed based on published definitions.

The detailed characteristics of the enrolled patients are summarized in Table 1. The median age was 43 years (range: 16–60). Seven patients (26.9%) had extramedullary disease at diagnosis, and 13 patients were classified as refractory or relapsed AML. At the time of transplantation, eight patients (30.8%) were in non‐remission (NR) or relapse status, while the remaining patients were in CR1 (*n* = 14) or CR2 (*n* = 4). Within 30 days post‐HSCT, neutrophil engraftment was achieved in all patients, and platelet engraftment was observed in 22 patients (84.6%). Five patients required thrombopoietin receptor agonist (TPORA) treatment for delayed platelet engraftment. Mucositis was the most common regimen‐related toxicity (80.7%), with most cases being well tolerated. Hematologic complete remission (CR) was achieved in 25/26 patients (96.2%), and 21/26 (80.8%) patients achieved Measurable Residual Disease (MRD) clearance at the day of hematopoietic reconstitution. The incidence of 100days acute GVHD and severe aGVHD was 34.6% and 15.4%, respectively.

**TABLE 1:** Basic characteristics of the enrolled patients.

	*N* = 26 (100%)
Age, median, range (years)	43 (16,60)
Sex, male	8 (30.8)
HCT‐CI, ≥ 2	10 (38.5)
WBC at diagnosis	23.6 (0.8–190)
Refractory/relapsed AML	13 (50)
Disease status at HSCT	
CR1	14 (53.8)
CR2	4 (15.4)
NR	8 (30.8)
Extramedullary disease	7 (26.9)
AML type, de novo AML/sAML	24(92.3)/−2 (7.7)
Chromosome karyotype	
Nomal	11 (42.3)
Monosomal karyotype	5 (19.2)
Complex karyotype	9 (34.6)
Unable to define the karyotype	1 (3.8)
Molecular biology	
FLT3‐ITD mutated	7 (26.9)
RUNX1 mutated	3 (11.5)
TP53 mutated	2 (7.7)
Mutated NPM1	4 (15.4)
KMT2A rearrangement	2 (7.7)
CSF3R mutated	3 (11.5)
BCOR Mutated	4 (15.4)
KRAS mutated	3 (11.5)
NRAS mutated	5 (19.2)
HSCT type	
MSD‐HSCT	10 (38.5)
HID‐HSCT	15 (57.7)
MUD‐HSCT	1 (3.8)
Times of chemotherapy pre‐HSCT, median (range)	4 (2,12)
Mean cell dose (range)	
NC × 10^8^/kg BW	11.8 (6.5∼21.8)
CD34+ × 10^6^/kg BW	4.7 (0.75∼11.0)

Abbreviations: BW, body weight (recipient); CR, Complete remission; HCT‐CI, hematopoietic cell transplantation comorbidity index; HID, haploidentical donor; HSCT, hematopoietic stem cell transplantation; MSD, matched sibling donor; MUD, matched unrelated donor; sAML, secondary AML.

**FIGURE 1:**
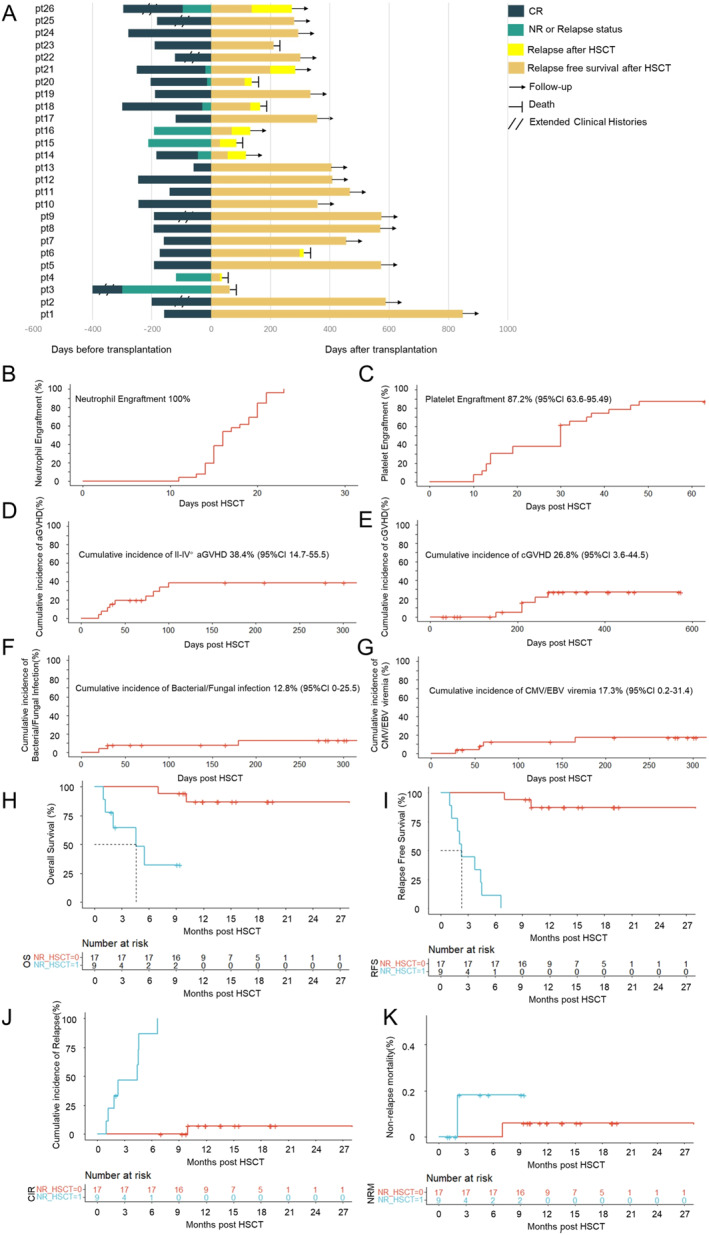
(A) Swimming plot showing the clinical outcomes of the 26 patients in this study with LIPO‐MIT involved conditioning regimen. (B) Cumulative incidence of neutrophil engraftment after allogeneic HSCT. (C) Cumulative incidence of platelet engraftment after allogeneic HSCT. (D) Incidence of II‐IV aGVHD after allogeneic HSCT. (E) Incidence of cGVHD after allogeneic HSCT. (F) Incidence of bacterial/fungal after allogeneic HSCT. (G) Incidence of CMV/EBV viremia after allogeneic HSCT. (H) OS of patients with CR1/CR2(blue lines) or NR/Relapsed disease (red lines). (I) RFS of patients with CR1/CR2(blue lines) or NR/Relapsed disease (red lines). (J) CIR of patients with CR1/CR2(blue lines) or NR/Relapsed disease (red lines). (K) NRM of patients with CR1/CR2(blue lines) or NR/Relapsed disease (red lines).

With a median follow‐up of 359 days (range: 30–848) post‐HSCT, nine patients experienced relapse, resulting in a 1‐year cumulative incidence of relapse (CIR) of 36.5 ± 9.81% (95% CI: 14.1%–53.1%). Two patients died due to transplant‐associated thrombotic microangiopathy (TA‐TMA) and severe pneumonia, contributing to a 1‐year NRM of 9.4 ± 6.34% (95% CI: 0%–21%). The 1‐year OS and RFS rates were 69.9 ± 9.76% (95% CI: 53.1%–91.9%) and 57.1 ± 9.82% (95% CI: 40.8%–80%), respectively. Among the seven patients with extramedullary disease, three relapsed (relapse incidence: 42.9%) at a median time of 132 days (range: 56–298) post‐transplantation, with only one patient experiencing extramedullary relapse. Key transplant outcomes are summarized in Figure 1 and Table S1.

We conducted a subgroup analysis based on disease status at transplantation (CR1/CR2 vs. NR/Relapse). No significant differences were observed in the incidence of grade II‐IV aGVHD (29.4% vs. 44.4%) or cGVHD (29.4% vs. 0%). However, 8/9 patients in NR/Relapsed cohort experienced relapse, with a median time to relapse of 100 days (range: 30–136). The remaining patient in this subgroup died of TA‐TMA on day 63. Patients transplanted in CR demonstrated significantly better outcomes. The 1‐year OS rates were 86.9% (95% CI: 71.4%–100%) for the CR1/CR2 group and 32.4% (95% CI: 10.8%–97.4%) for the NR/Relapse group. Similarly, the 1‐year RFS rates were 87.4 (95% CI: 72.4%–100%) and 0% for the CR1/CR2 and NR/Relapse groups, respectively. Notably, a difference was also observed in the CIR between the two groups (7.14% vs. 100%). Compared to transplantation performed in CR (including CR1 and CR2), transplantation during NR/relapse status appeared to be associated with a higher risk of relapse, inferior RFS, and worse OS.

The intensity of conditioning regimens has been demonstrated to influence outcomes in AMLs following allo‐HSCT. Recent discussions have focused on the incorporation of anthracycline agents, such as idarubicin (IDA), into the standard myeloablative BuCy regimen to enhance anti‐leukemic efficacy [8, 9, 10]. Mitoxantrone, a synthetic anthracycline with broad‐spectrum antitumor activity and a backbone of chemotherapy regimens for AML and lymphoma but constrained by cardiotoxicity. Encapsulating mitoxantrone within liposomes preserves its intrinsic pharmacological mechanism while mitigating adverse effects, such as cardiotoxicity, by ensuring liposomal integrity until the drug reaches the tumor tissue [5]. Compared to idarubicin‐containing regimens, our Lipo‐MIT protocol achieved comparable CR rates. Our findings indicate that LIPO‐MIT‐based conditioning is both safe and effective for high‐risk AML patients, albeit exclusively for those with controlled disease. As the first trial to add Lipo‐MIT into transplantation conditioning regimen, our study capitalizes on Lipo‐MIT's dual mechanism: attenuating systemic toxicity (e.g., cardiac, mucosal), while preserving antileukemic capacity via tumor‐selective biodistribution.

Nevertheless, it is crucial to recognize the limitations in this study, such as its limited sample size, and relatively short follow‐up. In summary, the outcomes of this investigation indicate favorable outcome following LIPO‐MIT involved regimens in patients achieving CR1/CR2 at transplantation.

## Author Contributions

**Xiaoyu Zhang:** performed the research, analyzed the data and wrote the paper. **Donglin Yang:** collected patients' data, managed the database and contributed essential reagents or tools and **Aiming Pang, Sizhou Feng** and **Mingzhe Han:** critically edited the manuscript. **Yi He** and **Erlie Jiang:** designed the research study, oversaw the research and critically reviewed the manuscript. All authors gave final approval for the manuscript.

## Ethics Statement

The patents were participants of a larger study, the NICHE‐BMT project (NCT04645199), which is the retrospective analysis of the IHCAMS recipients of bone marrow transplantation. This project was approved by the IHCAMS Clinical Research Academic Committee, and by the IHCAMS Ethics Committee on February 7, 2021 (IIT2021011‐EC‐1). All procedures were in accordance with the ethical standards of the institutional and national research committee (Ethics committee of Blood Disease Hospital, Chinese Academy of Medical Sciences IIT2021011‐EC‐1) and with the 1964 Helsinki Declaration and its later amendments or comparable ethical standards. The institutional review board approved all study procedures and forms.

## Consent

All participants signed written informed‐consent forms.

## Conflicts of Interest

The authors declare no conflicts of interest.

## Supporting information

Table S1

## Data Availability

All data generated or analyzed during this study are included in this published article.
