# Supplementary material for: The Efficacy and Safety of the Addition of Mitoxantrone Hydrochloride Liposome in Conditioning Regimen for High‐Risk Acute Myeloid Leukemia
Source: Hematol Oncol. 2025 Jul 3;43(4):e70116. doi: 10.1002/hon.70116 (PMC12226766; doi:10.1002/hon.70116)
Supplement: Supplementary file 1 — Table S1 [file HON-43-e70116-s001.docx]

**Supplementary Table 1. Efficacy and safety of the Lipo-MIT-involved regimen.**

|  | N=26 (100%) |
| --- | --- |
| Reconstitution Day |  |
| <5% blast | 25 (96.2%) |
| Complete MRD remission (<10^-2^%) | 21 (80.8%) |
| STR, median(range) | 98.4% (63.4%-100%) |
| One month after transplantation |  |
| <5% blast | 25 (96.2%) |
| Complete MRD remission (<10^-2^%) | 21 (80.8%) |
| STR, median(range) | 98.8% (10.8%-100%) |
| Neutrophil engraftment |  |
| Engraftment rate | 26 (100%) |
| Median (range) | 16 (11,23) |
| Platelet engraftment |  |
| Engraftment rate | 22 (84.6%) |
| Median (range) | 30 (10,48) |
| Infection |  |
| Bacteria/Fungus | 3 (11.5) |
| CMV | 1 (3.8) |
| EBV | 2 (7.6) |
| aGVHD |  |
| II | 5 (19.2) |
| III~IV | 4 (15.4) |
| cGVHD | 5 (19.2) |
| Mucositis |  |
| I~II | 16 (61.5) |
| III~IV | 5 (19.2) |
| Diarrhea |  |
| I~II | 9 (34.6) |
| III~IV | 1 (3.8) |
| Hepatic dysfunction |  |
| I | 1 (3.8) |

aGVHD: acute graft-versus-host disease; cGVHD: chronic graft-versus-host disease; MRD: minimal residual disease; CMV: cytomegalovirus; EBV Epstein-Barr Virus.
